# Modulation of calcium, callose synthesis, membrane permeability and pectin methyl-esterase activity affect cell wall composition and embryo yield during *Brassica napus* microspore embryogenesis

**DOI:** 10.1093/aob/mcaf054

**Published:** 2025-04-15

**Authors:** Antonio Calabuig-Serna, Daniel Sancho-Oviedo, Alba Rivas-Sendra, Estefanía Mata-Nicolás, Paloma Arjona-Mudarra, Ricardo Mir, Jose María Seguí-Simarro

**Affiliations:** Cell Biology Group, COMAV Institute, Universitat Politècnica de València, 46022 Valencia, Spain; Cell Biology Group, COMAV Institute, Universitat Politècnica de València, 46022 Valencia, Spain; Cell Biology Group, COMAV Institute, Universitat Politècnica de València, 46022 Valencia, Spain; Cell Biology Group, COMAV Institute, Universitat Politècnica de València, 46022 Valencia, Spain; Cell Biology Group, COMAV Institute, Universitat Politècnica de València, 46022 Valencia, Spain; Cell Biology Group, COMAV Institute, Universitat Politècnica de València, 46022 Valencia, Spain; Cell Biology Group, COMAV Institute, Universitat Politècnica de València, 46022 Valencia, Spain

**Keywords:** Androgenesis, *Brassica napus*, calcium, callose, cell wall, *in vitro* culture, *in vitro* embryogenesis, microspore embryogenesis, morphogenesis, pectin, pectin methyl-esterase

## Abstract

**Background and Aims:**

Microspore embryogenesis is a convenient inducible system to study the changes associated with the developmental reprogramming of cells. In this work, *Brassica napus* microspore cultures were used to study the role in the embryogenic switch of callose and pectin cell wall composition, which depends on Ca^2+^ levels.

**Methods:**

We used different chemicals to modulate Ca^2+^, callose and pectin methyl-esterification, including Ca(NO_3_)_2_, inositol 1,4,5-trisphosphate, 2-deoxy-d-glucose, benzyl alcohol, chitosan, epigallocatechin gallate and pectin methyl-esterase. Ca^2+^ distribution, callose and cellulose were imaged with FluoForte, Aniline Blue and Pontamine Fast Scarlet stainings, respectively, and observed with confocal microscopy.

**Key Results:**

Inhibition of callose synthesis with 2-deoxy-d-glucose demonstrated that callose is essential for induction of microspore embryogenesis. A moderate increase of Ca^2+^ levels with Ca(NO_3_)_2_ or inositol 1,4,5-trisphosphate promoted increased callose synthesis and deposition in the cell wall. However, the use of benzyl alcohol and chitosan to permeabilize the plasma membrane and allow for Ca^2+^ influx was not positive, because this prevented embryo development by inducing callus formation. Benzyl alcohol did not affect callose and cellulose deposition, but chitosan induced the formation of callose plugs, similar to those formed in response to pathogen attack. Inhibition of pectin methyl-esterase activity with epigallocatechin gallate during the first 3 days of culture produced ~70 % more embryos, but prolonged exposures were negative. Instead, increased pectin methyl-esterase activity during the first 3 days was not positive, but when applied for 7 days, embryos increased by ~60 %.

**Conclusions:**

Together, these results confirm the relevant role of calcium and callose during the first stages of microspore induction and suggest that the levels of pectin methyl-esterification in the cell wall are dynamic and that different cell wall compositions are required during the different stages of microspore embryogenesis.

## INTRODUCTION

*In vitro* culture of vacuolated microspores and young pollen grains, a process known as microspore embryogenesis, allows deviation of their development from the gametophytic to an embryogenic pathway to produce haploid and, eventually (upon genome doubling), doubled haploid (DH) embryos (Seguí-Simarro *et al.*, 2021*b*). This process accelerates the production of homozygous lines for hybrid seed production and constitutes an excellent model to study cell totipotency and the changes associated with the developmental switch.

At present, there are protocols available to induce this process in nearly 400 species (Seguí-Simarro *et al.*, 2021*a*). Exogenous factors, such as the induction and *in vitro* culture conditions, are key for the success of microspore embryogenesis (Seguí-Simarro *et al.*, 2021*b*). All the species need stress to become induced. For example, in *Brassica napus*, the induction treatment is a heat shock (HS) of 32 °C (Corral-Martínez *et al.*, 2021). Among the endogenous factors, the genotype of the donor plants is determinant. Different species have different levels of recalcitrance to induction, from moderate to absolute (Bueno *et al.*, 2003; Seguí-Simarro and Nuez, 2005; Corral-Martínez and Seguí-Simarro, 2012, 2014; Parra-Vega *et al.*, 2013; Seguí-Simarro, 2016; Rivas-Sendra *et al.*, 2020; Mir *et al.*, 2021). *Brassica napus* is a model for the study of this process because there are high-response genotypes, such as the DH4079 line, and low-response genotypes, such as the DH12075 line (Corral-Martinez *et al.*, 2020; Camacho-Fernández *et al.*, 2021). In both lines, induction of embryogenesis produces four types of structures with different embryogenic competence (Corral-Martinez *et al.*, 2020). There are highly embryogenic structures that produce the vast majority of embryos and include: (1) exine-enclosed (EE) structures (globular, compact, fully surrounded by exine), the most abundant highly embryogenic type; and (2) loose bicellular structures (LBS; two asymmetrically divided cells, sometimes with discrete exine breaks), which are less frequent than EE and soon differentiate into suspensor-bearing embryos (SUS). Conversely, barely embryogenic structures rarely produce embryos and include: (3) compact calli (CC; proliferative masses with broken exine) that never produce embryos; and (4) loose calli (LC; irregular masses of loosely attached cells with large areas without exine).

Apart from the genotype and the developmental stage of isolated microspores/pollen, another relevant endogenous factor is the level of intracellular calcium in its cationic form (Ca^2+^). Ca^2+^ is a universal signal for many different processes (Tian *et al.*, 2020). In microspore embryogenesis, Ca^2+^ levels are higher in the stages more responsive to induction, and after induction, Ca^2+^ levels increase even more during the first embryogenic stages; this is paralleled by callose deposition beneath the intine, which is one of the first cellular changes important for the developmental fate of the embryogenic microspore (Rivas-Sendra *et al.*, 2017, 2019). Increasing extracellular Ca^2+^ levels with Ca(NO_3_)_2_ or intracellular Ca^2+^ levels with inositol 1,4,5-trisphosphate (InsP_3_) increased embryo yield, whereas chelation of extracellular Ca^2+^ with EGTA or intracellular Ca^2+^ with BAPTA-AM reduced embryo yield (Calabuig-Serna *et al.*, 2025). Thus, there seems to be a link between Ca^2+^ levels, callose synthesis and embryo yield.

Another approach to increase intracellular Ca^2+^ levels and callose deposition has been the use of digitonin, known to increase Ca^2+^ uptake through membrane permeabilization (Waldmann *et al.*, 1988). Digitonin promoted callose deposition in the subintinal layer of *B. napus* embryogenic microspores (Rivas-Sendra *et al.*, 2019), reinforcing the link between increased Ca^2+^ levels and callose deposition. Thus, we explored the effects of using other plasma membrane-permeabilizing agents, such as benzyl alcohol (BA) and chitosan. BA (α-hydroxytoluene) increases plasma membrane fluidity, making it more permeable to ions and small molecules (Horváth *et al.*, 1998; Sangwan *et al.*, 2001). In plants, BA activates a heat-induced Ca^2+^-permeable ion channel in response to increased plasma membrane fluidity, therefore promoting a Ca^2+^ influx and triggering an HS response (Saidi *et al.*, 2009). Indeed, BA was able to trigger embryogenesis in *B. napus* microspores in a similar manner to HS (Pauls *et al.*, 2006). Chitosan is derived from chitin, the main component of fungal haustoria and insect exoskeletons and a strong elicitor of plant anti-pathogen response. Chitosan elicits several events, including the disturbance of plasma membrane stability, the increase of its fluidity and the displacement of membrane-bound Ca^2+^ (Siegel and Daly, 1966; Young *et al.*, 1982), which promotes callose synthesis (Pearce and Ride, 1982) as a physical barrier against pathogens. Despite the similarities of these treatments to HS, their effects in the cell walls of embryogenic microspores have scarcely been studied.

Ca^2+^ also has a structural role relevant for other cell wall components, such as pectin. Pectin is transported from Golgi stacks, via vesicles, in a highly methyl-esterified form and is locally de-esterified by pectin methylesterases (PMEs; Bou Daher and Braybrook, 2015). Then, de-esterified pectin molecules cross-links to each other and interact with Ca^2+^, forming a pectate gel that imparts rigidity to the cell wall (Micheli, 2001). In *B. napus* embryogenic microspores, the PME-like gene (*BnPME*) is expressed at low levels during the first developmental stages, allowing for high levels of methyl-esterified pectin (Solis *et al.*, 2016). In turn, the cell walls of highly embryogenic structures are characterized by high levels of highly methyl-esterified pectin, which confer them flexibility for growth and expansion (Camacho-Fernández *et al.*, 2021). Thus, there seems to be a link between pectin and induction of embryogenesis, suggesting that modulation of pectin methyl-esterification could influence the efficiency of microspore embryogenesis.

In this work, we studied the role of Ca^2+^-dependent callose and pectin. We used different chemicals to modulate their levels and studied the effect on embryo yield. For callose, we used 2-deoxy-d-glucose, an inhibitor of callose biosynthesis (Radford *et al.*, 1998), and Ca(NO_3_)_2_ and InsP_3_ to increase extracellular and intracellular Ca^2+^ levels, respectively. We also evaluated the effect of permeabilizing plasma membranes with BA and chitosan in callose and cellulose deposition in the newly formed cell walls. To modulate pectin composition, we used epigallocatechin gallate (EGCG) to inhibit PMEs and therefore increase the levels of highly methyl-esterified pectin, and PME to reduce these levels. Our results show that altering cell wall composition at the level of the components studied affects embryo yield. With methyl-esterified pectin, it is even possible to improve yield.

## MATERIALS AND METHODS

### Plant material

The *B. napus* high-response DH line DH4079 was used for all the experiments. Plants were grown in 20 cm pots in a growth chamber with a 16 h–8 h photoperiod and at 20 °C during their vegetative growth period. Upon blooming, plants were transferred to chambers at 10 °C with the same photoperiod. Flower buds were collected ≥1 week after transference.

### Microspore culture

Microspore cultures were done as described by Corral-Martínez *et al.* (2021). Flower buds were collected at the right developmental stage for their microspores to be induced to embryogenesis. Flower buds were surface sterilized with 70 % ethanol (for 30 s) and 10 % bleach solution (for 10 min). Then, they were rinsed three times in sterile distilled H_2_O and poured into sterile glass beakers with NLN-13 medium (Nitsch and Nitsch, 1967; Duchefa, The Netherlands) supplemented with 130 g/L sucrose, pH 5.8. Microspores were released from buds by crushing with a sterile syringe piston in NLN-13. The microspore suspension was filtered through a 41 µm nylon filter and centrifuged (100*g*, 4 min, 4 °C). The supernatant was decanted, and pelleted microspores were resuspended in 10 mL of fresh NLN-13 medium. This process was repeated twice, and after the third centrifugation the pelleted microspores were resuspended in 1 mL of NLN-13 medium to estimate microspore density using a improved Neubauer chamber as described by Camacho-Fernández *et al.* (2018). The final volume was adjusted by adding NLN-13 medium up to a density of 40 000 microspores/mL, and 1 mL of suspension was plated in 3-cm-wide sterile culture dishes. The induction treatment consisted of a 32 °C HS for 3 days, after which dishes were transferred to 25 °C, always in darkness. Embryo yield was measured by counting the total number of embryos per dish after 1 month in culture.

### Fluorescence microscopy

FluoForte (Enzo Life Sciences) staining was used to detect Ca^2+^ in microspores as described by Rivas-Sendra *et al.* (2019). Briefly, microspores were centrifuged (4 min, 200*g*, room temperature), resuspended in phosphate-buffered saline (PBS) and centrifuged again. Pelleted cells were resuspended in equal volumes of PBS and 0.2 g/L FluoForte solution, incubated for 30 min (in darkness), washed with PBS, centrifuged once more (2 min, 200*g*), and mounted in microscope slides with Mowiol anti-fading mounting solution (17 % Mowiol 4-88 + 33% glycerol in PBS). Samples were observed in a Nikon E1000 epifluorescence microscope. For callose and cellulose staining, samples were collected at defined stages, as described in the Results, and fixed overnight at 4 °C with 4 % paraformaldehyde in PBS (pH 7.4), washed three times with PBS, then stored at 4 °C in 0.1 % paraformaldehyde in PBS until use. Callose was stained with 0.1 % Aniline Blue (AB; Fluka) in PBS for 20 min. For BA and chitosan experiments, callose staining was preceded by staining with 10 µg/mL propidium iodide in PBS (PI; Fluka) for 10 min and three washes with PBS. Samples were mounted in 17 % Mowiol 4-88 (Sigma-Aldrich) and 33 % glycerol (v/v) in PBS. Cellulose was stained with 0.01 % Pontamine Fast Scarlet (S4B) in PBS for 30 min, washed three times with PBS, mounted in a 50 %:50 % mixture of Mowiol and 2.5 μg/mL 4′,6-diamidino-2-phenylindole (DAPI; Sigma-Aldrich), then incubated for 15 min. Samples stained only with DAPI were prepared by mounting them directly in the Mowiol + DAPI mixture. All the samples were incubated in darkness and observed with a ZEISS 780 Axio Observer confocal laser scanning microscope. DAPI and Aniline Blue were excited with 405 nm and PI and S4B with 561 nm laser lines. Emission was recorded between 450–490 and 580–650 nm, respectively. Images were processed with Zeiss Efficient Navigation (ZEN) proprietary software and post-processed with FIJI software (Schindelin *et al.*, 2012). For Ca^2+^, callose and cellulose staining, ≥20 different individual structures per stage were studied.

### Chemical treatments

Ca(NO_3_)_2_ was added to cultures as an additional source of Ca^2+^. To release intracellular Ca^2+^, inositol 1,4,5-trisphosphate (InsP_3_) was used. 2-Deoxy-d-glucose was used to inhibit callose biosynthesis. Benzyl alcohol (BA) and chitosan were used to increase plasma membrane permeability. All chemicals, including EGCG, were purchased from Sigma-Aldrich, except for Ca(NO_3_)_2_ (Duchefa), and pectin methylesterase 8A *Dickeya dadantii* for pectin modulation (NZYTech). Stocks (pH 5.8) were prepared according to the manufacturer’s specifications, dissolved in distilled H_2_O except for chitosan, prepared in 1 % acetic acid, stirring it overnight at 4 °C. Stocks were sterilized by filtration through 0.22 μm filters and stored at −20 °C. The corresponding volumes of each stock were added to microspore cultures to reach the working concentration described in the Results. Controls were prepared by adding to cultures the same volumes of the corresponding solvent. In general, compounds were added at the time of culture initiation (day 0) and removed after 3 days, 7 days or 1 month (continuous exposure), which is when cultures in all cases were finished and embryos counted. For each experiment, a minimum of three biological replicates were prepared. Embryo yield typically shows variability among cultures. For convenience when comparing among treatments, results were expressed after normalizing the embryo yield of controls to the reference value of 100, then calculating the corresponding values of the treatments. One-way ANOVA (*P* ≤ 0.05) was performed to determine statistical differences among culture conditions. Then, significance groups were established through the least significance difference (LSD) method.

## RESULTS

**Fig. 1.**
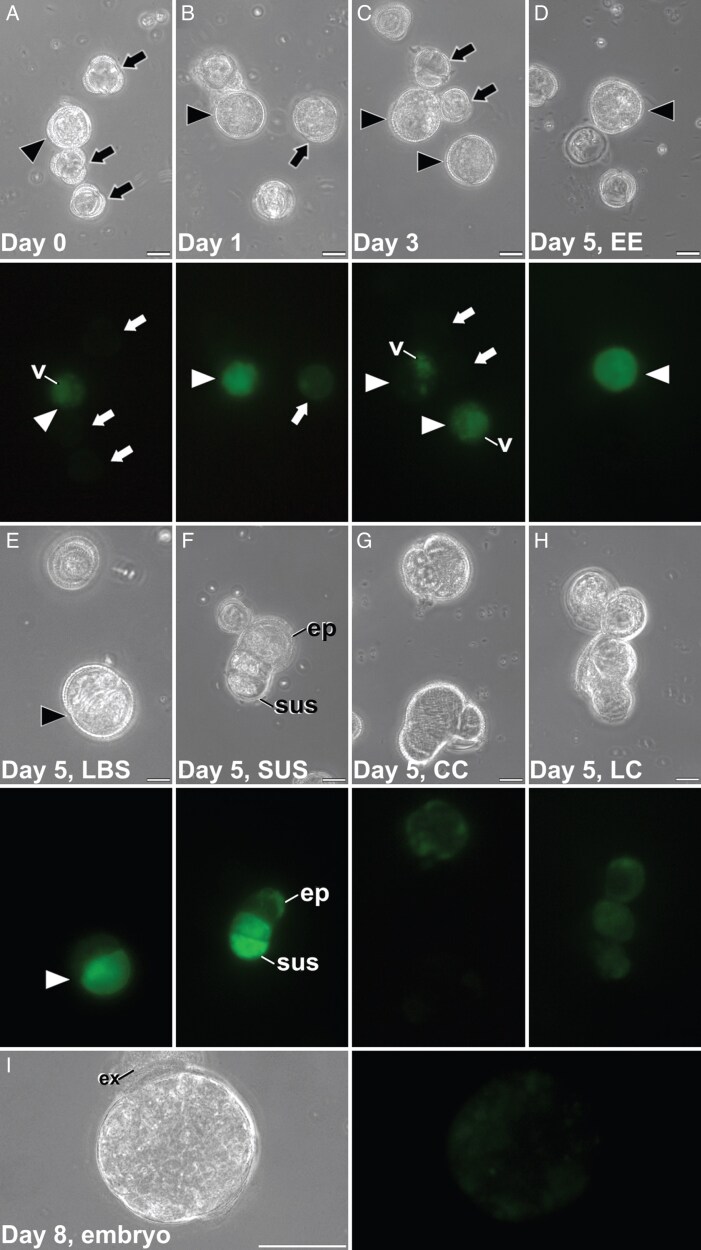
*Brassica napus* microspore cultures and Ca^2+^ detection with FluoForte. Paired images of the same microscopic field imaged by phase contrast optics (top image) and epifluorescence (bottom image). (A) Freshly isolated, vacuolated microspores at day 0. The arrowhead points to a vacuolated microspore with visible FluoForte signal, principally in the vacuole (v). Microspores at younger stages (arrows) show no FluoForte staining. (B) One-day-old culture showing an enlarged, growing microspore (arrowhead) with an intense FluoForte signal, and a non-growing microspore (arrow) with a less intense signal. (C) Three-day-old induced structures with visible FluoForte signal (arrowheads) concentrated in vacuoles (v), together with arrested microspores (arrows) with no FluoForte staining. (D–H) Five-day-old cultures showing an exine-enclosed (EE) structure (arrowhead in D), an LBS still mostly covered by exine (arrowhead in E) and transformed into an early suspensor (SUS) embryo (F), two compact callus (CC; G) and a loose calli (LC) structure (H). (I) Microspore-derived globular embryo. Abbreviations: ep, embryo proper; ex, exine; sus, suspensor. Scale bars: 20 µm in A–H; 50 µm in I.

Microspore cultures at the optimal stage for induction of embryogenesis were composed principally of vacuolated microspores and young pollen grains, together with a few microspores at other, non-inducible stages (Fig. 1A). FluoForte staining was visible in vacuolated microspores, as previously described (Rivas-Sendra *et al.*, 2017; Calabuig-Serna *et al.*, 2025), whereas in other, younger stages, the FluoForte signal was barely detectable. In 1-day-old cultures (Fig. 1B), induced microspores were characterized by their enlargement, although no evident structural differences among the different types of embryogenic structures could be detected. The FluoForte signal was bright in growing, enlarged microspores, as opposed to other microspores, where growth was not evident and FluoForte staining was much less bright or even null. In three-day-old cultures (Fig. 1C), growing embryogenic microspores also exhibited intense FluoForte signals. In five-day-old cultures (Fig. 1D-H), it was already possible to identify clearly the four types of embryogenic structures described to be induced from *B. napus* microspores (Corral-Martinez *et al.*, 2020; Camacho-Fernández *et al.*, 2021, 2024). They include compact EE structures with intense FluoForte signal (Fig. 1D), LBS (Fig. 1E) that developed into SUS embryos with a different FluoForte signals in their embryo proper and suspensor domains (Fig. 1F), and disorganized CC (Fig. 1G) and LC (Fig. 1H) callus-like structures, with loosely connected cells where exine is partly or fully detached and FluoForte signal is still detectable. In eight-day-old cultures (Fig. 1I), fully grown microspore-derived globular embryos, after bursting from the exine in all cases, presented very low, barely detectable Ca^2+^ levels, similar to those of non-induced microspores. We used this system to test the effect on microspore embryogenesis of the chemicals described next.

### *Modulation of callose deposition with 2-deoxy-* *d* *-glucose, Ca(NO*_*3*_)_*2*_  *and InsP*_*3*_

**Fig. 2.**
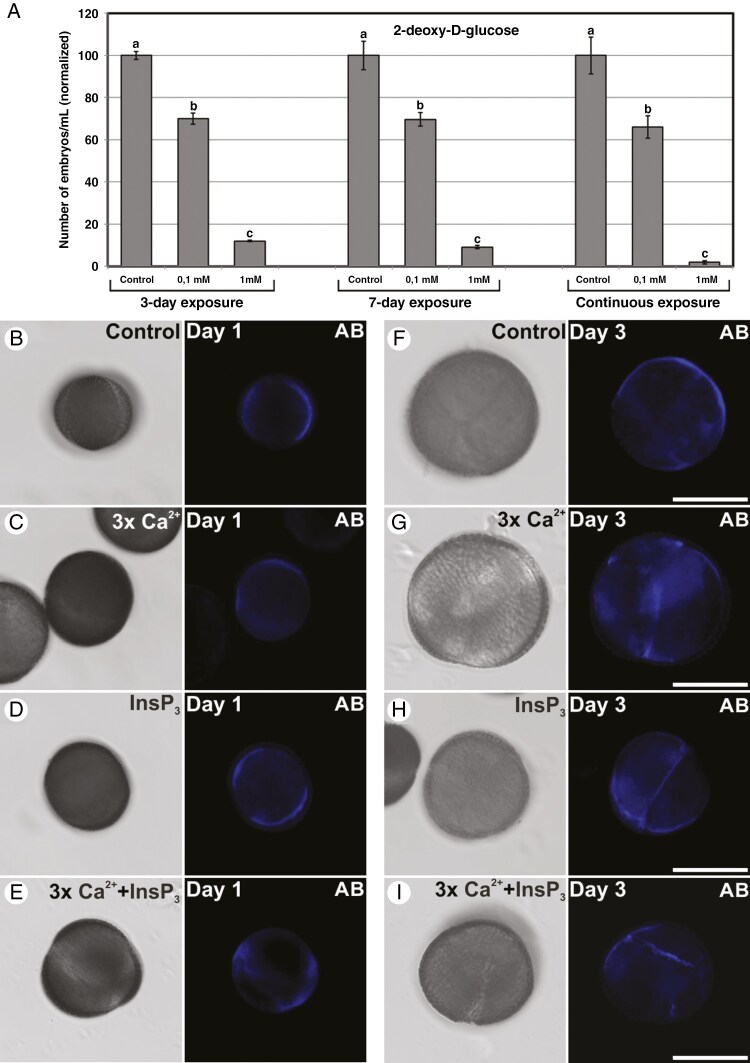
Callose deposition in embryogenic microspores. (A) Effect of inhibiting callose biosynthesis with 0.1 and 1 mm 2-deoxy-d-glucose during the first 3 days of culture, during 7 days and continuously. Effects are expressed as the number of embryos produced per millilitre of culture medium, normalizing control values to 100. Different letters indicate significant differences according to the LSD test (*P* ≤ 0.05). (B–I) Paired images of the same microscopic field imaged by phase contrast optics (left image) and fluorescence (right image) to reveal Aniline Blue staining of callose in 1-day-old (B–E) and 3-day-old (F–I) microspores cultured in standard conditions (control, B, F), with 3 × Ca(NO_3_)_2_ (3 × Ca^2+^; C, G), 10 µm InsP_3_ (InsP_3_; D, H) and 3 × Ca(NO_3_)_2_ combined with 10 µm InsP_3_ (3 × Ca^2+^ + InsP_3_; E, I). Scale bars: 20 µm.

To study the role of callose in microspore embryogenesis, we applied to cultures 2-deoxy-d-glucose, an inhibitor of callose biosynthesis, during the first 3 days, 7 days and continuously, and we calculated embryo yield in each case (Fig. 2A). A significant and dose-dependent reduction of embryo yield was observed clearly with both 0.1 and 1 mm concentrations, which proves the need for callose deposition to produce viable microspore-derived embryos. The effect, however, was not time dependent, because 3-day, 7-day and continuous exposures produced very similar profiles of embryo yield compared with controls. This indicated that the main effect of 2-deoxy-d-glucose in callose inhibition is produced during the first 3-day stage. Together, these results suggest that callose deposition is necessary for induction of microspore embryogenesis and further embryo development, but the main role of callose during microspore embryogenesis is restricted to the first 3 days of the process, the stage when microspores are induced to embryogenesis.

Next, we explored the possible relationship between Ca^2+^ and callose deposition, focusing on the initial 3-day stage mentioned above. It is known that increasing Ca^2+^ levels by adding either 3 × Ca(NO_3_)_2_ or 10 µm InsP_3_ during the first 3 days of culture is sufficient to increase the final yield of microspore-derived embryos (Calabuig-Serna *et al.*, 2025). Given that callose synthases are Ca^2+^ dependent, we checked whether the positive effect of Ca^2+^ is related to a Ca^2+^-dependent increase in callose deposition, detected by Aniline Blue staining. We cultured microspores exposed during 3 days to 3 × Ca(NO_3_)_2_, 10 µm InsP_3_, and a combination of both (Fig. 2B–I). One day after initiation of *in vitro* culture (Fig. 2B–E), no cell divisions were generally observed yet, and Aniline Blue principally stained the subintinal layer, described to be synthesized at this stage (Parra-Vega *et al.*, 2015). In comparison to control, no visible differences in callose staining were observed among Ca(NO_3_)_2_, InsP_3_ and combined treatments. At day 3 of culture (Fig. 2F–I), callose staining was slightly more intense in the 3 × Ca(NO_3_)_2_ and 10 µm InsP_3_ treatments than in the controls, being observed at both the subintinal layer and the inner cell walls formed in dividing cells. However, no clear differences were found in Aniline Blue staining between these two treatments, nor between controls and the combined 3 × Ca(NO_3_)_2_ + 10 µm InsP_3_ treatment. In summary, callose deposition is essential for microspore embryogenesis, and Ca^2+^ levels artificially increased with Ca(NO_3_)_2_ or InsP_3_ induce a slight increase in the levels of callose deposition in the cells of embryogenic microspores.

### Effect of BA on cellulose and callose deposition

**Fig. 3.**
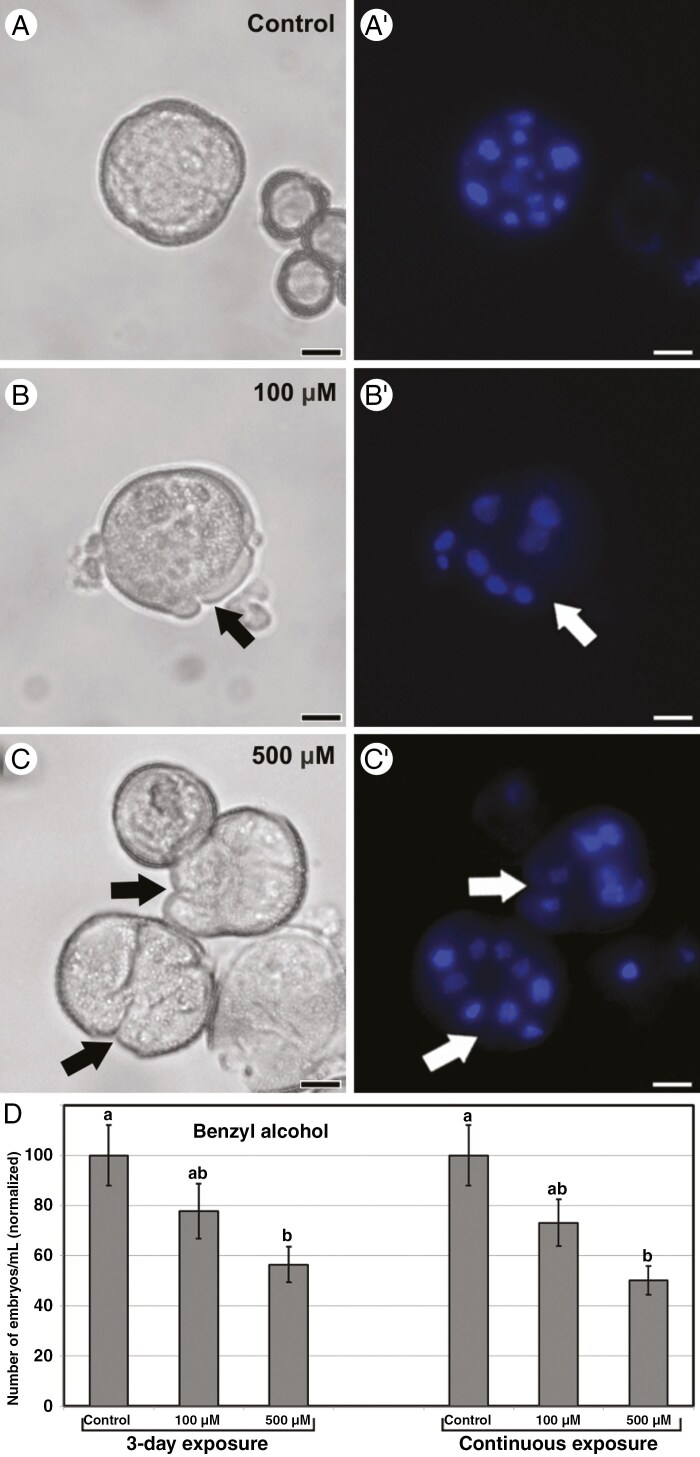
Effect of BA in microspore embryogenesis. (A–C′) Representative embryogenic structures formed after 6 days of culture in control cultures (A, A′) and in cultures with 100 μm (B, B′) and 500 μm BA (C, C′). (A–C) Phase contrast images. (A′–C′) Epifluorescence microscopy images of DAPI-stained samples. Arrows point to regions with ruptured exine. Scale bars: 10 µm. (D) Effects on embryo yield of adding different concentrations of BA during the first 3 days of culture and continuously. Effects are expressed as the number of embryos produced per millilitre of culture medium, normalizing control values to 100. Different letters indicate significant differences according to the LSD test (*P* ≤ 0.05).

We added BA at 100 and 500 μm to microspore cultures in order to increase Ca^2+^ uptake through membrane fluidization (Saidi *et al.*, 2009). Compared with controls (Fig. 3A, A′), both 100 (Fig. 3B, B′) and 500 μm BA (Fig. 3C, C′) promoted the occurrence of compact (CC) and loose (LC) callus-like disorganized multicellular structures, with loosely attached cells and large regions devoid of exine, equivalent to those described as barely embryogenic structures (Corral-Martinez *et al.*, 2020). Consistent with this, embryo yield was negatively affected by exposure to BA in a dose-dependent manner (Fig. 3D). The negative effect on embryo yield was remarkably similar in 3-day and continuous exposures to BA, which suggests that this effect is exerted principally during the initial stage of embryogenesis induction.

**Fig. 4.**
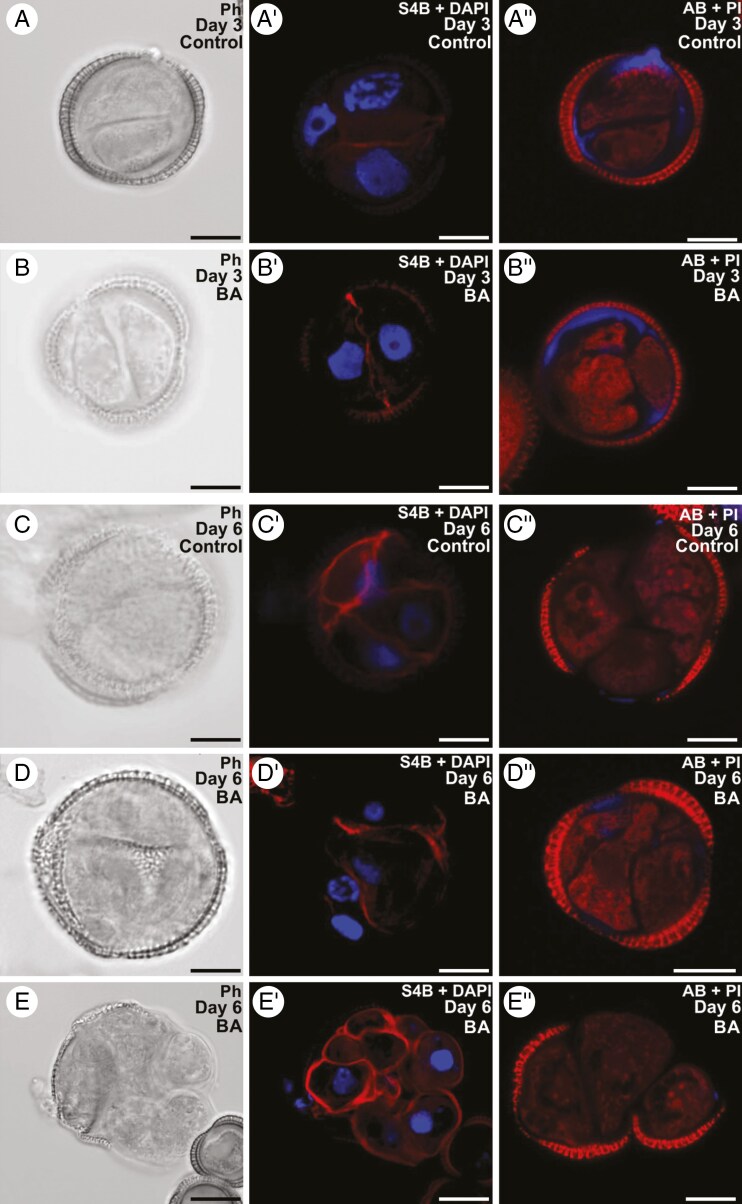
Effect of BA in cellulose and callose deposition during microspore embryogenesis. Structures formed in *Brassica napus* microspore cultures treated with BA 500 μm. (A–E) Phase contrast (Ph) images. For cellulose detection (A′–E′), cellulose (red) is stained with S4B, and DAPI (blue) is used to stain nuclei for contrast. For callose detection (A″–E″), callose (blue) is stained with Aniline Blue (AB), and propidium iodide (PI, red) is used for contrast. (A–A″) Three-day-old dividing microspore in control culture without BA. (B–B″) Three-day-old dividing microspore in cultures treated with BA. (C–C″) Six-day-old exine-enclosed embryogenic structure in control culture without BA. (D–D″) Six-day-old exine-enclosed (EE) embryogenic structure in cultures treated with BA. (E–E″) Six-day-old compact callus (CC) structure in cultures treated with BA. Scale bars: 10 µm.

Next, we tested whether BA could affect the patterns of cellulose and callose deposition in the cell walls. As in controls (Fig. 4A–A″), S4B staining of cellulose in 3-day-old structures exposed to BA (Fig. 4B–B″) revealed no detectable cellulose in the subintinal layer, and a weak and discontinuous deposition in the thin inner cell walls. Callose was detected by Aniline Blue staining in inner cell wall and subintinal layer regions, mainly where they join. At the 6-day stage, differences with respect to controls (Fig. 4C–C″) were not noticeable in any type of structure. In highly embryogenic structures (principally EE structures; Fig. 4D–D″), cellulose was found in incomplete inner cell walls and in discrete areas beneath the intine, whereas callose presence was limited to scarce residues in the subintinal layer and rarely in inner cell walls. Likewise, barely embryogenic structures (CC and LC), with loosely connected cells and large regions devoid of exine (Fig. 4E–E″), showed a continuous cellulose wall surrounding each individual cell and almost no callose. In conclusion, treatment with BA had a negative effect on embryo induction, whereas callose and cellulose deposition was unaffected.

### Effect of chitosan on cellulose and callose deposition

**Fig. 5.**
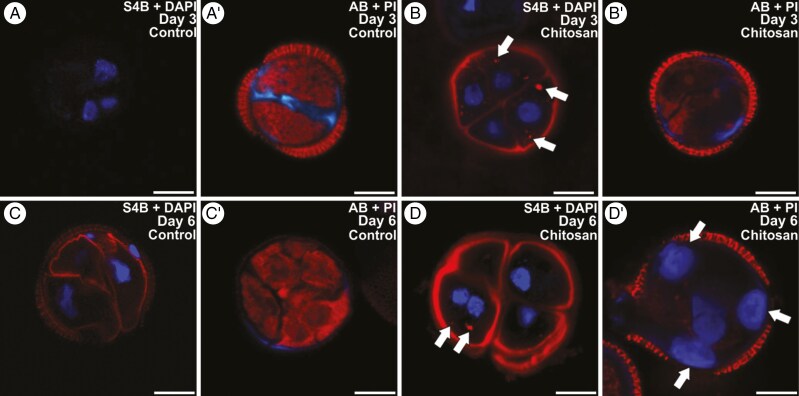
Effect of chitosan in cellulose and callose deposition during microspore embryogenesis. Structures formed in *Brassica napus* microspore cultures treated with chitosan 6.6 nm. For cellulose detection (A–D), cellulose (red) is stained with S4B, and DAPI (blue) is used to stain nuclei for contrast. For callose detection (A′–D′), callose (blue) is stained with Aniline Blue (AB), and propidium iodide (PI, red) is used for contrast. (A, A′) Three-day-old dividing microspores in control culture without chitosan. (B, B′) Three-day-old dividing microspores in cultures treated with 6.6 nm chitosan. Arrows point to small cellulose cytoplasmic spots. (C, C′) Six-day-old embryogenic structures in control culture without chitosan. (D, D′) Six-day-old embryogenic structures in cultures treated with 6.6 nm chitosan. Arrows point to small cellulose (D) and large callose (D′) cytoplasmic spots. Scale bars: 10 µm.

Chitosan (0.66, 6.6 and 66 nm) was also used to increase membrane fluidity and therefore intracellular Ca^2+^ levels. With respect to untreated control cultures, where cellulose deposition was almost absent and callose was abundant in the subintinal layer and inner cell walls (Fig. 5A, A′), no differences were observed with 0.66 nm. In turn, cells were unable to develop in any way with 66 nM. The only relevant differences were observed with 6.6 nm. In general, 6.6 nm chitosan reduced the embryogenic response and favoured the development of callus-like, disorganized structures. At the 3-day stage, cellulose was abundant in all inner and outer walls, surrounding all cells and in small cytoplasmic spots (Fig. 5B). In general, callose was scarce, present only in some fragments of the subintinal layer, and rarely observed in inner cell walls (Fig. 5B′). As previously described (Parra-Vega *et al.*, 2015), controls at the 6-day stage presented embryogenic structures where cellulose was abundant and callose was thoroughly removed from all cell walls (Fig. 5C, C′). However, in 6-day-old embryogenic structures treated with chitosan, S4B staining showed a thick and intensely stained cellulose wall entirely surrounding each cell, together with cytoplasmic spots (Fig. 5D), and callose staining revealed unusual, compact and well-delimited plugs of callose, especially in callus-like structures (Fig. 5D′). In summary, chitosan severely reduced callose synthesis in the cell walls and accelerated cellulose synthesis. However, the most remarkable effect was the induction of abnormal patterns of ectopic deposition for both polysaccharides as cytoplasmic deposits.

### Modulation of pectin methyl esterification

**Fig. 6.**
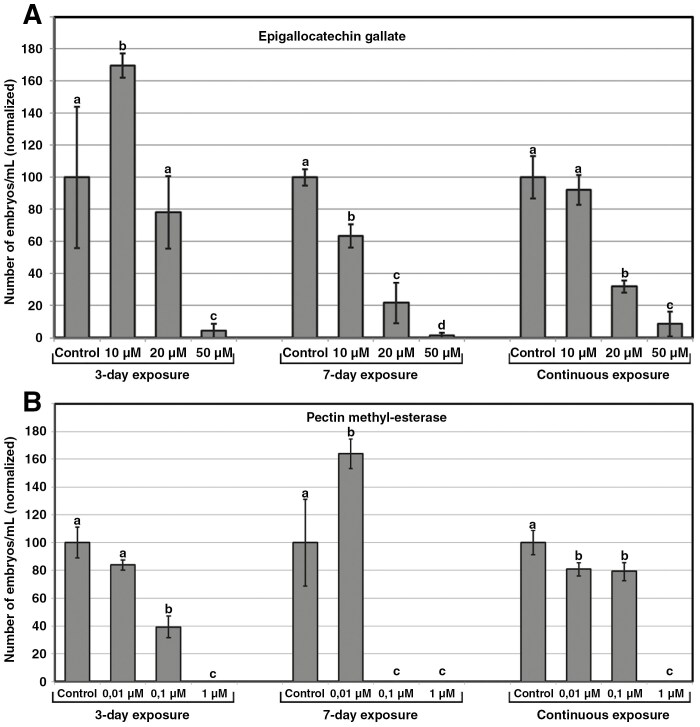
Effects in microspore embryogenesis of modulating pectin methyl-esterase activity. (A) Effect of inhibiting pectin methyl-esterase activity with the addition of EGCG to the culture medium. (B) Effect of adding exogenous pectin methyl-esterase activity. For A and B, chemicals and concentrations were applied during the first three days of culture, during seven days, and continuously. Effects are expressed as number of embryos produced per mL of culture medium, normalizing control values to 100. Different letters indicate significant differences according to the LSD test (*p* ≤ 0.05).

The levels of pectin esterification have also been related to external Ca^2+^ supply (Wu *et al.*, 2018), in addition to the embryogenic fate (Camacho-Fernández *et al.*, 2021). Given that pectin is delivered in a methyl-esterified form to the cell walls, where it is locally de-esterified by PMEs (Bou Daher and Braybrook, 2015), we studied the role of methyl-esterified pectin in microspore embryogenesis by modulating their levels. First, we used EGCG, a natural inhibitor of PME activity (Lewis *et al.*, 2008), in order to increase the levels of methyl-esterified pectin. EGCG was applied to cultures at different concentrations and exposure times. As seen in Fig. 6A, inhibition of PME activity for 3 days with 10 µm EGCG had a very positive effect on embryo yield, which increased ~70 %. No significant effect was found with 20 µm, and 50 µm almost suppressed embryo production. With 7-day exposures, all concentrations showed a dose-dependent negative effect on embryo yield. With continuous exposures, 10 µm had no significant effect on embryo yield, and the rest of concentrations showed a dose-dependent negative effect. Thus, the only positive effect of inhibiting PME activity was observed when microspores were exposed to 10 µm EGCG during the first 3 days of culture, when microspores are induced to switch to embryogenesis.

Finally, we added PME to the cultures in order to reduce the levels of methyl-esterified pectin (Fig. 6B). With 3-day exposures, 0.01 µm did not show any significant effect, and higher concentrations showed a dose-dependent negative effect on embryo yield. However, 7-day exposures with 0.01 µm PME showed a clear positive effect on embryo yield, which increased by ~60 %, whereas higher concentrations nearly suppressed embryo production. This is logical for 1 µm, because it also suppressed embryo yield for 3-day and continuous exposures, but not for 0.1 µm, which reduced but did not suppress embryo yield in these cases. We believe that there was some sort of technical problem with this specific concentration and exposure time that did not affect the rest of the assays. With continuous exposures, all concentrations had a negative effect on embryo yield, with the highest producing no embryos. These results, together with those of EGCG, show that modulation of PME activity, hence the levels of pectin methyl-esterification, has an impact in embryo yield. Depending on the exposure time, both decreasing and increasing PME activity might be positive to produce more microspore-derived embryos.

## DISCUSSION

### Increased Ca^2+^ levels promote callose deposition, which is essential for induction of embryogenesis

In this work, we showed that callose deposition is essential during the 3-day stage of embryogenesis induction, when Ca^2+^ levels increase, because inhibition with 2-deoxy-d-glucose precludes embryo production. Similar results were found in *Arabidopsis* and carrot somatic embryogenesis, where callose deposition was described as essential for proper induction of somatic embryos (Godel-Jedrychowska *et al.*, 2020; Calabuig-Serna *et al.*, 2023*a*, *b*). Indeed, the formation of a transient callose layer surrounding only embryogenic cells is consistently observed in many different *in vitro* embryogenesis systems, including somatic (Maheswaran and Williams, 1985; Helleboid *et al.*, 1998; You *et al.*, 2006; Grimault *et al.*, 2007; Godel-Jedrychowska *et al.*, 2020; Calabuig-Serna *et al.*, 2023*a*, *b*) and microspore-derived embryogenesis (Parra-Vega *et al.*, 2015; Rivas-Sendra *et al.*, 2019; Camacho-Fernández *et al.*, 2021), being considered a mechanism to isolate embryogenic cells from the surrounding environment (Dubois *et al.*, 1991).

We also showed that increasing Ca^2+^ levels with either Ca(NO_3_)_2_ or InsP_3_, known to enhance embryo yield (Calabuig-Serna *et al.*, 2025), promoted a slight increase in callose deposition in 3-day-old embryogenic microspores (Fig. 2G, H). However, the combined use of Ca(NO_3_)_2_ + InsP_3_ does not produce such a positive effect on callose deposition (Fig. 2I) or on embryo yield (Calabuig-Serna *et al.*, 2025), which confirms the existence of a maximum threshold, beyond which increased Ca^2+^ levels are not positive. It was proposed that increased Ca^2+^ levels would increase the amount of microspores reaching the minimum Ca^2+^ levels needed for induction (Calabuig-Serna *et al.*, 2025). Our results extend this notion to promote callose deposition in individual cells. In conclusion, one of the roles of increased Ca^2+^ levels during induction of microspore embryogenesis would be to activate callose biosynthesis, essential for successful induction.

### Benzyl alcohol promotes callus formation and prevents embryo development

The impact of Ca^2+^ on callose deposition is in line with previous results with digitonin, a membrane-permeabilizing agent that, at low concentrations, increased callose deposition in *B. napus* microspore cultures (Rivas-Sendra *et al.*, 2019). In this work, we used other membrane fluidizing drugs, BA and chitosan. Although previous, preliminary data on BA in microspore cultures were slightly positive (Parra-Vega *et al.*, 2015), this study, more exhaustive, shows that the resulting structures are largely disorganized, which precludes their transformation into embryos. This is consistent with the digitonin data and with other works on the negative effect of BA in embryo production when applied together with a 32 °C HS (Pauls *et al.*, 2006). This report also showed that at 25 °C, without HS, BA slightly increased embryo yield, because it ‘mimics’ the membrane fluidization produced by HS, but at a much lower level. Thus, when combining BA + HS, the small effect of BA is negligible compared with the stronger effect of HS. This correlates with our results, where 100 μm BA + HS produced no significant effects on embryo yield or on callose and cellulose deposition, and 500 μm was detrimental only in terms of embryo yield.

### Chitosan appears to elicit callose synthesis related to pathogen attack response

Chitosan is also known to increase plasma membrane fluidity and therefore Ca^2+^ influx (Siegel and Daly, 1966; Young *et al.*, 1982). In microspore cultures, chitosan induced the occurrence of callus-like structures, with absent or defective cell walls, abnormal cellulose deposition and cytoplasmic callose plugs (Fig. 5). This was somewhat unexpected, because a beneficial effect on embryo yield was reported previously (Ahmadi and Shariatpanahi, 2015). We applied a long treatment, hence the observed effects are probably attributable to long-term toxicity. Indeed, previous shorter treatments with higher doses showed similarly negative effects, causing high callus formation (Ahmadi and Shariatpanahi, 2015). Anyway, the most striking result was the observed pattern of reduced wall deposition and formation of large cytoplasmic plugs at days 3 and 6, remarkably different from other callose-inducing compounds. As part of the pathogenesis-related (PR) response, chitosan produces rapid and transient membrane depolarization (Amborabé *et al.*, 2008), which causes H^+^ and Ca^2+^ influx which, in turn, elicits callose synthesis (Pearce and Ride, 1982). The PR response also includes chitinase synthesis to fight against pathogens. Interestingly, several chitinases, among other enzymes involved in the PR response, were found to be strongly upregulated in induced barley anther cultures (Jacquard *et al.*, 2009), whereas developing maize microspore-derived embryos were found to excrete two chitinase isoforms into the culture medium (Borderies *et al.*, 2004).

As seen, there are interesting coincidences between the induction of both the PR response and microspore embryogenesis. It seems that cultured microspores perceive heat and chitosan as stressors and react by triggering general plant defence mechanisms, which would include chitinase and callose synthesis. CalS12/GSL5 is a Ca^2+^-dependent callose synthase involved in the PR response and in callose deposition upon wounding (Jacobs *et al.*, 2003), and it is present across all the stages of microspore/pollen development, with its main function being the synthesis of the transient callose walls in tetrads after meiosis (Enns *et al.*, 2005). Given that CalS12/GSL5 is also expressed in *B. napus* embryogenic microspores, where a role in the synthesis of the subintinal layer was proposed (Rivas-Sendra *et al.*, 2019), this enzyme could be a good candidate to be activated by HS-related Ca^2+^ influx and, in a stronger and more unspecific way, by chitosan.

### PME activity precludes the induction of embryogenic structures

Conventional cell walls are major Ca^2+^ stores, because de-esterified pectin has the ability to bind enormous amounts of Ca^2+^ (Wu *et al.*, 2018) to become the stiff Ca^2+^-pectate gel that provides rigidity to the cell wall (Micheli, 2001). However, cell wall composition is dynamic and adapts to changing cell demands. For example, the first stages of microspore embryogenesis in *B. napus* are characterized by low expression levels of the pectin methylesterase-like gene (*BnPME*) and high levels of methyl-esterified pectin, whereas later stages of embryo development showed the opposite scenario (Solis *et al.*, 2016). Thus, one could reasonably predict that inhibition of PME in microspore cultures would reduce pectin de-esterification, thereby keeping pectin in its highly methyl-esterified form and decreasing cell wall stiffness. This, in turn, would promote microspore embryogenesis, because a high level of highly methyl-esterified pectin is a feature of cell walls of highly embryogenic structures that gives them the wall flexibility needed for initial cell growth and expansion and for eventual exine rupture (Camacho-Fernández *et al.*, 2021). This was confirmed by the use of EGCG to keep pectin highly methyl-esterified. Three-day inhibition of PME activity with 10 µm EGCG produced ~70 % more embryos. This could possibly be related to the prevention of the HS-derived cell wall stiffness. HS exposure activates PMEs and also increases their de-esterification rate, which helps to develop heat tolerance through the formation of rigid Ca^2+^-pectate gels with low methyl-esterified pectin (Wu *et al.*, 2018). However, prolonged exposures to EGCG produced no positive results, which reinforces the notion that the positive effect of inhibiting PMEs occurs during the first stages of embryogenesis induction, when microspores must have a plastic, flexible cell wall to transform into embryogenic structures. By day 7 of culture and beyond, embryogenic structures are already formed, cell growth has burst exines, and the levels of low-methyl-esterified pectin are progressively higher (Corral-Martínez *et al.*, 2019; Camacho-Fernández *et al.*, 2021).

The results obtained with the addition of PME were, in general, opposite to those of inhibiting PME, and therefore aligned with the reasoning above. An excess of PME activity during the first 3 days, when highly methyl-esterified pectin favours the development of highly embryogenic structures, would not be positive for embryo yield. Instead, at day 7, when multicellular structures show cell walls with more conventional composition, increased cell wall stiffness and adhesion might increase the number of embryos developing successfully. In turn, continuous exposure to increased PME activity throughout embryo development would produce an excess of de-esterified pectin incompatible with embryo development. In conclusion, altering PME activity during the first stages of microspore embryogenesis has severe consequences for the final embryo yield.

### Concluding remarks

These results, together with previous works (Calabuig-Serna *et al.*, 2025), demonstrate the remarkable impact of Ca^2+^ levels in cell wall dynamics in terms of changes in callose and pectin composition during the first days (the inductive stage) of microspore embryogenesis. These changes are key for the final fate of microspores, directly impacting on embryo yield, and have the potential to be used to explore new ways for improving the efficiency of microspore embryogenesis in recalcitrant species. In particular, increasing intracellular Ca^2+^ levels was shown to be beneficial to improve embryo yield (Calabuig-Serna *et al.*, 2025). However, not all possible strategies have proved useful. We hereby show that membrane permeabilization to facilitate Ca^2+^ intake is detrimental. Maybe these treatments allow for the entry of excessive Ca^2+^, reaching toxic levels, or maybe Ca^2+^ is triggering other, unwanted cellular responses that negatively affect embryogenesis, as we showed for chitosan. In either case, it must be noted that HS alone already destabilizes the plasma membrane (Wu *et al.*, 2018). Additional treatments to increase Ca^2+^ levels should not destabilize it further.

## Data Availability

All data supporting the findings of this study are available within the paper.
