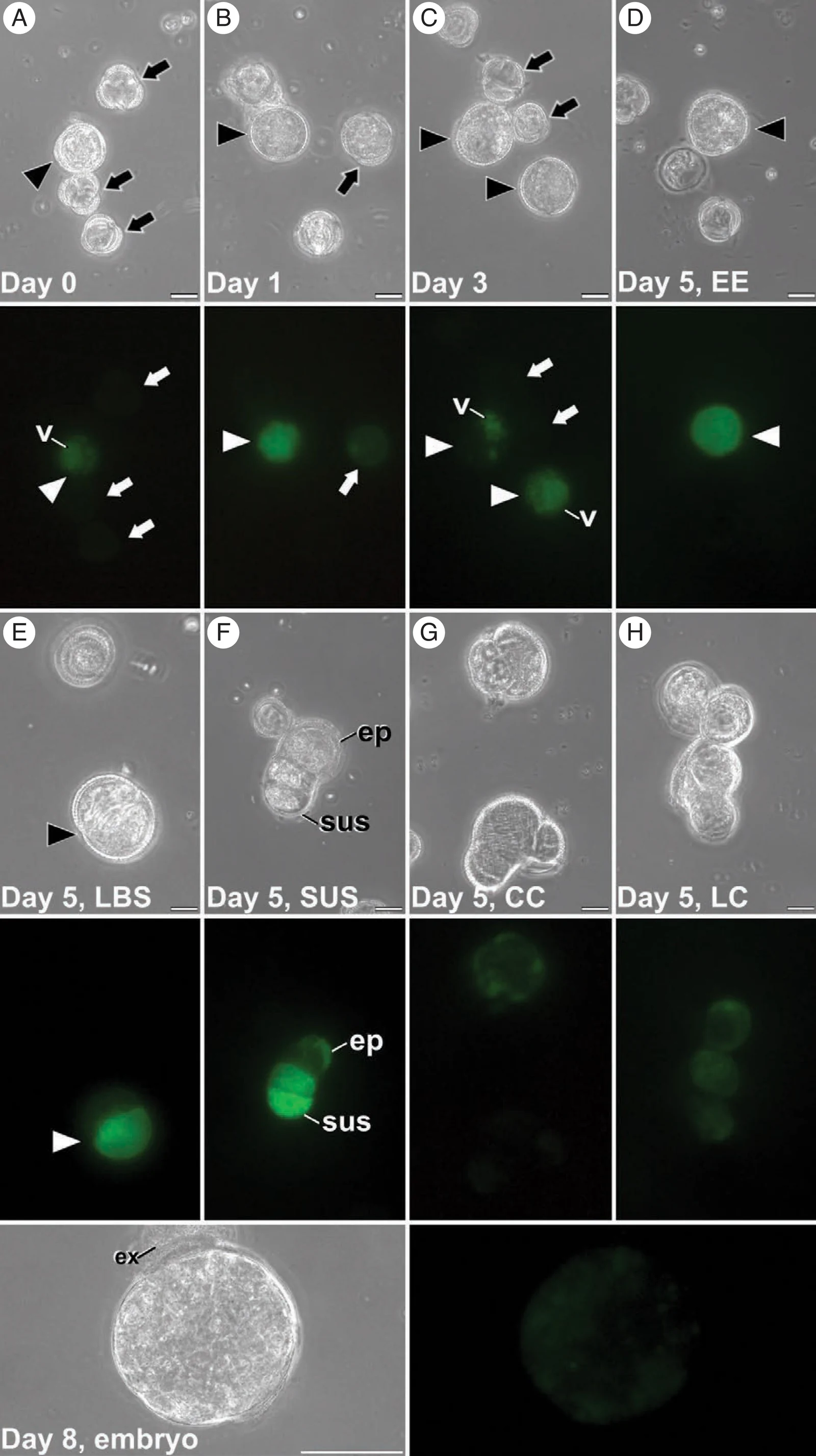# Correction to: Modulation of calcium, callose synthesis, membrane permeability and pectin methyl-esterase activity affect cell wall composition and embryo yield during *Brassica napus* microspore embryogenesis

**DOI:** 10.1093/aob/mcag017

**Published:** 2026-02-03

This is a correction to: Antonio Calabuig-Serna, Daniel Sancho-Oviedo, Alba Rivas-Sendra, Estefanía Mata-Nicolás, Paloma Arjona-Mudarra, Ricardo Mir, Jose María Seguí-Simarro, Modulation of calcium, callose synthesis, membrane permeability and pectin methyl-esterase activity affect cell wall composition and embryo yield during *Brassica napus* microspore embryogenesis, *Annals of Botany*, Volume 136, Issue 1, July 2025, Pages 99–110, https://doi.org/10.1093/aob/mcaf054

In the originally published version of the manuscript, there were errors introduced into Figure 1 during production process in the format of the lettering of images. These have been corrected in the article. Figure 1 should read:

**Figure:**
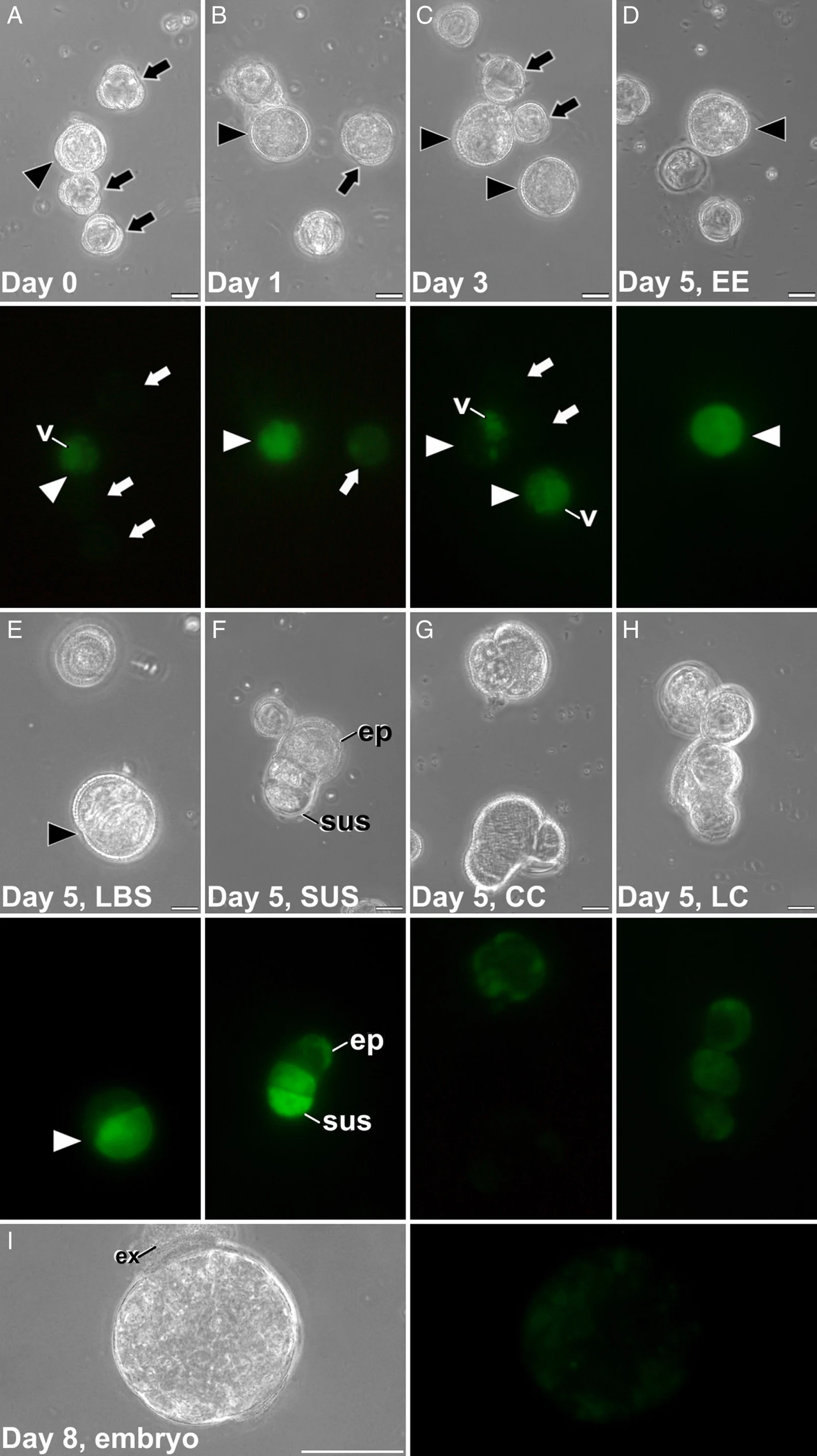


instead of:

**Figure:**